# The relationship of dental caries and dental fear in Malaysian adolescents: a latent variable approach

**DOI:** 10.1186/1472-6831-14-19

**Published:** 2014-03-12

**Authors:** Rashidah Esa, Ai Leng Ong, Gerry Humphris, Ruth Freeman

**Affiliations:** 1 Department of Community Oral Health & Clinical Prevention, Faculty of Dentistry, University of Malaya, 50603 Kuala Lumpur, Malaysia; 2 Dental Department, Ministry of Health, Bayan Baru, Penang, Malaysia; 3 Bute Medical School, University of St Andrews, Fife, Scotland, UK; 4 Dental Health Services Research Unit, University of Dundee, Dundee, Scotland, UK

**Keywords:** DMFT, Dental fear, Adolescents

## Abstract

**Background:**

To investigate the role of geography (place of residence) as a moderator in the relationship between dental caries disease and treatment experience and dental fear in 16-year-olds living in Malaysia.

**Methods:**

A multi-stage-stratified sampling method was employed. Five hundred and three, 16-year-olds from 6 government secondary schools participated in this study. The questionnaire examined participants’ demographic profile and assessed their dental fear using the Dental Fear Survey (DFS). The clinical examination consisted of the DMFT as the outcome measure of dental caries disease and treatment experience by a single examiner (ICC = 0.98). Structural equation modelling inspected the relationship between dental fear and dental caries disease and treatment experience.

**Results:**

The mean DMFT was 2.76 (SD 3.25). The DT, MT and FT components were 0.64 (SD 1.25), 0.14 (SD 0.56) and 1.98 (SD 2.43) respectively. Rural compared with urban adolescents had significantly greater mean numbers of decayed and missing teeth. The mean DFS score was 40.8 (SD 12.4). Rural compared with urban adolescents had significantly higher mean scores for physical symptoms of dental fear. The correlation between dental fear (DFS) and dental caries disease and treatment experience (DMFT) was 0.29, p < 0.0001. The structural equation model fitted the raw data well (*χ*^2^ = 9.20, df = 8, p = 0.34). All components of DMFT were closely associated in equal strength to the unidimensional hypothetical latent variable of dental caries disease and treatment experience. The strength of the relationship between dental fear and dental caries disease and treatment experience varied in accordance with place of residence.

**Conclusion:**

In conclusion a relationship between dental fear and dental caries disease and treatment experience was shown to exist in 16-year-old adolescents living in Malaysia. This study showed that the rural–urban dichotomy acted as a moderator upon this relationship.

## Background

The prevalence of tooth decay among Malaysian adults is 90% [1-4] with over ten teeth on average being affected. Moreover those residing in rural compared with urban areas have greater prevalence of active decay disease experience. These observations are considered to be discouraging as The School Dental Service (SDS) in Malaysia has been in existence since the 1950’s and in 1985 evolved into a comprehensive dental health care service for schoolchildren. The aim of the service being to ensure that schoolchildren are dentally-fit at the end of their school education [5] and to achieve this aim the SDS provides incremental dental care for all up to 17 years of age. The SDS offers both oral health preventive and dental treatment services. The preventive activities within the schools include dental health education talks, dental exhibitions, tooth brushing programmes, and fissure sealant applications. Dental treatment includes the provision of scale and polishes, fillings and tooth extractions. Oral health service delivery is via the school dental clinics located within the school precinct or mobile dental team using mobile dental units or mobile dental clinic in a bus or caravan. The mobile dental units are mainly used within the rural areas.

Despite having this readily accessible dental health care service, there remains a high prevalence of decay into dentine and missing teeth in the adult population particularly in rural areas [1-4]. What is the possible explanation for this observation? Could it reflect reduced accessibility to dental care associated with place of residence? Could it be associated with Malaysian school-leavers experiencing not only physical (e.g. geography) but also psychological (e.g. dental anxiety) barriers to accessing dental treatment? [6-8]. Adopting Cohen’s accessibility factor framework it maybe suggests that a possible barrier to accessing dental treatment is dental anxiety associated with previous frightening dental treatment experiences. Is it possible that Malaysian school-leavers avoided dental treatment because of dental fear? In support of this latter suggestion, a satisfaction survey with the SDS among 438, 16-year-old adolescents, Othman and Jaafar [9] found that fear of dental treatment was of central importance with regard to avoiding dental treatment and non-acceptance of treatment provided was associated with extreme dental fear in another group of Malaysian primary schoolchildren [10].

A relationship between frightening dental treatment experience and dental anxiety has been proposed in the literature [8,11-13] with some theorists suggesting that a relationship between dental caries disease and treatment with dental fear also exists [11,13-22]. Some have suggested a positive relationship whereas others have shown a negative association between dental fear and dental caries and treatment experiences [14,19-22]. A possible explanation for the lack of consensus is that all previous studies adopted raw variables in their analysis without adjusting for measurement error. This inevitably will produce lower associations due to attenuation. The construction of latent variables and employment of structural equation modelling enables the testing of associations of “true” variables with errors partialled out [23]. Hence, a positive methodological development can be introduced to assist the study of the relationship between dental fear and dental caries and treatment experience.

Although dental fear has been extensively studied worldwide, limited studies are available in Malaysia. It is imperative that in the SDS, some effort is directed towards recognizing and helping dentally anxious children and adolescents cope with their dental anxiety so that later in life they are able to access dental services without worry or fear.

Moreover, the role of location in the study of dental disease is a feature that is also poorly investigated in Malaysia. It may be suggested that ease of access may be somewhat more favourable in urban areas and that non-attendance in urban localities will be less compared with rural areas due to geographical location. Therefore reduced attendance at rural dental clinics for preventive treatments and an increased attendance for pain-only treatments could result in increased dental fear. Consequently, the association between dental anxiety and dental caries disease and treatment experience it may be proposed may be less in urban surroundings in comparison to rural areas. Hence geography or place of residence could act as a moderator of the relationship between dental anxiety and dental caries disease and treatment experience. The above proposed methodology will assist in this regard. Therefore the aim was to investigate the role of geography (place of residence) as a moderator in the relationship between dental caries disease and treatment experience and dental fear in 16-year-olds living in Malaysia.

## Methods

### Sample

The participants were 16-year-olds from the 3 main ethnic groups: Malays, Chinese and Indians residing and attending schools in urban and rural areas in the South-West District of Penang Island. This district is less well developed and is known for its agricultural produce and is one of the five administrative districts of the island and mainland Penang State. The rural areas are easily accessible by road. A multi-staged stratified sampling method was employed. The schools were divided into urban and rural in the first stage, according to the classification by the local Education Department. All schools (two urban and four rural) in the South-West District were included. Two residential schools were excluded because majority of the students resided in other Districts. Participants whose parents had signed the consent forms for the SDS were included in the final sample (98.5%). However, twenty-four participants were excluded due to their parents’ refusal to the dental treatment provided by the SDS.

There were 1564 children of which 675 and 889 resided in urban and rural areas respectively. The Malays and Chinese students were stratified according to their gender and were selected by proportionate sampling from class lists. As there were only 91 Indian students all were included in the final sample. The minimum sample size was calculated based on the single proportion formula [24] and the age specific caries prevalence from the National Oral Health Survey of schoolchildren 2007 [25]. An additional 20% was included to increase the response rate. The minimum estimated sample size was 444. The estimated sample size was further increased to include all the children selected from the class lists.

### Questionnaire

The 20-item Dental Fear Survey (DFS) of Kleinknecht was used. The measure comprises three factors: avoidance of dental treatment (behavioural), somatic symptoms of anxiety (physiological) and anxiety caused by dental stimuli (feelings), with an additional single question on dental fear in general [17,26,27]. The items are rated on a 5-point Likert scale with equal weights given to all items with scores ranging from 20 (no dental fear) to 100 (extreme dental fear). The questionnaire was translated into “Bahasa Melayu” (Malay version) and back translated into English. The questionnaire was self-administered in the classroom.

### Dental examination

The examination used DMFT [28] as a measure of disease and treatment experience. The D component was conceptualised as obvious decay experience. Obvious decay experience included caries at the pulpal (severe decay) and visual dentinal (established decay) levels as well as at the enamel level to improve recording of the disease threshold. The treatment experience was assessed through the F component of the DMFT as the care index (F/DMFT%) as well as through the M component (M/DMFT%). Prior to data collection, the dental examiner was calibrated and an assessment of intra-examiner reliability (single examiner) was conducted. The intra-class correlation coefficient (ICC) value was 0.98. The training of examiner was conducted in the dental school against a benchmark examiner. During the survey, duplicate examination was done on 5% of the participants examined daily. This subsample was randomly selected by the dental scribe. The intra-examiner reliability was high with an ICC value of 0.99.

The dental examination took place once the self-administered questionnaire was completed in another room using a portable dental chair and a portable standard artificial illumination. Throughout the data collection period two dental scribes were present.

### Ethical considerations

Ethical approval was obtained from the Ethical Committee, Faculty of Dentistry, University of Malaya, prior to the conduct of the study. Approval to conduct the study was also obtained from the Ministry of Education, the State School Director and the principals of all participating schools.

### Statistical methods

All data were entered into SPSS v15. Analyses were performed including frequency breakdown of categorical variables, and means (SDs) statistics derived for continuous variables. Total scale scores were assumed to behave as interval scales. Internal consistencies of the dental fear sub-scales were inspected using Cronbach’s alpha [avoidance of dental treatment (0.71), somatic symptoms of anxiety (0.70) and anxiety caused by dental stimuli (0.90)]. Mann–Whitney U-tests were calculated for comparing means. A structural equation model was designed to inspect in detail the relationship between dental fear and dental caries disease and treatment experience [29-31]. The model consisted of two latent factors, namely dental caries disease and treatment experience (defined by the three variables: number of decayed, missing and filled teeth) and dental fear (defined by the three subscales: Avoidance of dental care; Somatic symptoms and Dental stimuli). Each of the two sets of variables incorporated an error term (i.e. disturbance) enabling the disattenuated or ‘true’ correlation to be observed. The success of specifying each variable of the dental fear and dental caries disease and treatment experience latent variables could be examined by checking the ‘factor loadings’ from the latent variables to their respective indicators. Approximate equality in these values would support the use of the latent variable approach and not require additional models for separate indicators to be run. Maximum likelihood estimation was utilized to prepare parameter estimates using AMOS v17 enabling the calculation of the saturated model solution and further models fitted to inspect the moderating effect of adolescents’ place of residence (urban versus rural). Multi-group SEM was applied to test for group equivalence of the estimated covariance between the latent factors of dental fear and dental caries experience across place of residence. This testing was applied by running the unconstrained model and constrained model (i.e. covariance set to be equal across the two groups) simultaneously and the resulting chi-square statistics compared. A small non-significant difference between the overall model fit chi-square values would indicate equivalence between the group pair (i.e. rural and urban). Maximum likelihood method provides indices of overall goodness of fit including overall Chi Square, Comparative Fit Index (CFI) and Root Mean Square Error of Approximation (RMSEA) [32]. The latter 2 indices are conventionally set at > 0.95 and < 0.06 respectively for an indication of reasonable fit of the raw data to the proposed model. The parameter estimate divided by their standard error provides the critical ratio (CR) which for an alpha level of 0.05 will be less than or equal to 1.96. Bootstrap estimates (2000 samples drawn) were calculated to derive unbiased confidence intervals [33] for the final model. This was supplemented by the Bollen-Stine bootstrap procedure [34] to test the overall bias due to the use of maximum likelihood in this study with samples that may show skewed distributions. That is, the model Chi-square is adjusted for distributional misspecification. All statistical tests were 2 sided and alpha was set at 0.05.

A power analysis using the method of MacCullum, Browne and Sugawara [35] was conducted to determine the appropriate sample size required for the SEM model with a given power of 0.80 at a significance level of 0.05 to test the hypothesis of perfect fit (null hypothesis Ha: RMSEA = 0.00) versus moderate fit (Ha: RMSEA = 0.06). With degrees of freedom set at 8 for the measurement model the number of cases were calculated to be approximately 520.

## Results

A total of 518 sixteen-year-old adolescents were selected from the selected schools class lists. However, 15 participants did not attend during the data collection period. The response rate was 97.1% and 503 participants completed the questionnaire and oral examination. There were more rural participants as compared to urban (1.5: 1) whilst the proportion of females (53.5%) to males (46.5%) was almost equal.

The Dental Fear Survey (DFS) scores ranges from 20 to 79; the mean score for all participants was 40.8 (SD 12.43). Table 1 examines the variation of the DFS mean component scores across residence. A statistically significant difference was found for mean scores for somatic symptoms of anxiety between adolescents residing rural (8.86 ± 2.94) and adolescents residing in urban (8.05 ± 2.62) areas. There were no significant differences demonstrated in mean scores for avoidance of dental treatment or dental anxiety caused by dental stimuli by place of residence.

**Table 1:** Comparison of adolescents’ mean dental fear subscales scores by place of residence

	**Adolescents: urban localities (n = 200)**	**Adolescents: rural localities (n = 303)**	**z**	**p**
Scale 1: avoidance of dental care				
Mean (sd)	2.77 (1.30)	2.91 (1.57)	1.12	0.27
Scale 2: somatic symptoms				
Mean (sd)	8.05 (2.62)	8.86 (2.94)	3.15	0.002
Scale 3: dental stimuli				
Mean (sd)	28.64 (10.09)	30.08 (9.61)	1.61	0.11

The mean DMFT for all adolescents was 2.76 (SD 3.25). The DT, MT and FT components were 0.64 (SD 1.25), 0.14 (SD 0.56) and 1.98 (SD 2.43) respectively. The D component contributed 23.20% and the M component contributed 5.40% of the mean DMFT. The F component and hence the care index was 71.70%. Table 2 shows the mean number of decayed (DT), missing (MT) and filled (FT) teeth by place of residence. Rural adolescents had a significantly higher mean DMFT as compared to urban adolescents. For the urban adolescents 13.10% of the DMFT was explained by the D component, 2.30% by the M component and 83.70% by the F component. In rural adolescents 27.10% of the DMFT was explained by the D component, 6.30% by the M component and 66.20% explained by the F component. The mean DT and MT were statistically significantly higher for rural than urban adolescents (Table 2).

**Table 2:** Comparison of adolescents dental caries disease and treatment experience (DMFT) by place of residence

	**Adolescents: urban localities (n = 200)**	**Adolescents: rural localities (n = 303)**	**z**	**p**
Decayed teeth (DT)				
Mean (sd)	0.30 (0.62)	0.86 (1.49)	−4.86	<0.001
Missing teeth (MT)				
Mean (sd)	0.05 (0.24)	0.20 (0.68)	−2.52	<0.05
Filled teeth (FT)				
Mean (sd)	1.81 (2.36)	2.10 (2.47)	−1.61	0.11
Dental caries disease and treatment experience				
(DMFT)				
Mean (sd)	2.16 (2.69)	3.17 (3.52)	−3.34	<0.001

The fit of the structural equation model for the total sample was excellent as indicated by the small chi-square =9.20, df = 8, *p* =0.325; and fit statistics: CFI = 0.997 and RMSEA = 0.017; 95% CI: 0.000 to 0.057. The standardized parameter estimates are presented in Table 3 and are equivalent to factor loadings. They show the decayed, missing and filled variables have virtually equal explanatory value to describe the latent variable of ‘dental caries disease and treatment experience’. In addition, the equality of these factor loadings supports our view that this latent variable is specified uniformly by the three indicators: DT, MT and FT. Somatic symptoms and dental avoidance subscales relate strongly to the latent factor ‘dental fear’. The ‘dental stimuli’ variable relates somewhat less strongly. The overall correlation between the two latent variables was 0.292, exhibiting a high critical ratio (CR) of 3.711, *p* < 0.0001).

**Table 3:** Standardized parameter estimates (maximum likelihood) of factor loadings from structural equation model of total sample

	**Latent factors**	
	**Dental caries disease and treatment experience**	**Dental fear**
**Indicator variables**		
Decayed teeth (DT)	0.532	
Missing teeth (MT)	0.526	
Filled teeth (FT)	0.528	
Avoidance of dental treatment		0.746
Somatic symptoms		0.747
Dental stimuli		0.596

Note: all estimates significant p < 0.001.

The boot strapping procedure (Bollen-Stine) showed a similar *p* value of 0.369 to that associated with the maximum likelihood chi-square reported above (i.e. *p* = 0.325). This result indicated that the raw data were not systematically deviating from assumptions of normal distributions.

SEM enables the investigator to fix parameters to be set to be equal across specified samples. To test the equivalence of the model fit across the two geographic groups, namely: rural and urban it was found that constraining the covariance between dental fear and dental caries experience across place of residence did result in a higher chi-square value (chi-square difference = 5.76, df = 1, *p* < 0.05). This demonstrated that the correlations were significantly different across adolescents’ place of residence (Table 4).

**Table 4:** Comparison of structural equation models

***Model comparison***	***χ***^***2***^	***df***	***CFI***	***RMSEA***	***Δ χ***^***2 #***^	***df***	**p**
Rural/Urban					5.758	1	0.016
Unconstrained	10.668	16	1.00	0.000			
Constrained	16.426	17	1.00	0.000			

# difference in chi square between nested models.

On inspection it was found that the rural sample exhibited a statistically significant correlation of 0.308 (CR = 2.99, *p* = 0.003) between dental caries disease and treatment experience and dental fear whereas for urban it was 0.163 (CR = 1.44, *p* = 0.150) which was not significant.

## Discussion

This study aimed to investigate the role of geography (place of residence) as a moderator in the relationship between dental caries disease and treatment experience and dental fear in 16-year-olds living in Malaysia. A structural equation model was designed to inspect in detail the relationship between dental fear and dental caries disease and treatment experience.

The mean score for dental caries disease and treatment experience was 2.76 with 71.70% percent of the DMFT being composed of filled teeth. Differences in the prevalence of decayed and missing teeth were noted between urban and rural populations. Rural adolescents had significantly greater mean numbers of decayed and missing teeth. While all of the adolescents had carious teeth filled or extracted, differences were noted in the extent of the type of treatment experience. The differences, for instance, in the proportion of the DMFT explained by the M component in adolescents residing in rural compared with urban areas implied that in addition to a restorative intervention, those in rural localities also had greater surgical (teeth extracted) intervention. Some support for this proposition may be gleaned from a comparison of the Care Index which was 66.20% in adolescents residing in rural areas compared to 83.70% for those from urban areas. This finding implies that restorative intervention was greater in urban compared with rural localities. Previous epidemiological surveys of 16-year-old schoolchildren also provides additional evidence since the treatment of dental caries was different with regards to the provision of restorations and extraction of teeth in those populations residing in rural compared with urban localities [25].

Adolescents from rural localities were more fearful of dental treatment than their urban counterparts. A possible explanation for increased dental fear, in rural areas may be due to the different types of dental treatment received [6,8] and/or due to the differences in the type of dental service provided [9,10]. For instance mobile dental units provide dental services to rural schools whereas in urban schools, dental clinics are located within the school vicinity. Moreover, when asked over a third of rural adolescents were dissatisfied with the dental services provided by the mobile dental units [36].

A somewhat unexpected finding was the virtual equal contribution of each component to the constructed latent variable of obvious caries experience. It is important to note that this analysis refers specifically to the nature of the relationships between the single indicators, that is DT, MT and FT, and not the average levels of each indicator. The possibility that each indicator provides the same contributory power requires replication with other samples, however researchers may find reassurance that each aspect of the formal clinical examination is important for understanding the associations of obvious caries experience with other psycho-social and demographic factors.

Nevertheless this investigation shows that a link exists between dental caries disease and treatment experience and dental fear with geography acting as a moderator. The robustness of the model appears to be reasonable as it was not influenced extensively by fluctuations of non-normal distributions as shown by the bootstrapping procedure. While the direction of effect may be questioned by some, it would seem to us that regardless of the direction of the effect, we have presented strong evidence for the relationship between dental fear and dental caries disease and treatment experience. The strength of this relationship between urban and rural samples was not uniform. Evidence was found for a stronger association in the rural participants than urban adolescents. This suggests that a social gradient with regard to treatment experience may be proposed. The notion of a social gradient (from urban to rural) is implied by the increased proportion of the DMFT being composed of missing teeth in adolescents residing in rural localities compared with an increased Care Index in those adolescent residing in urban areas in Malaysia [3,4]. The adoption of multiple groups SEM had enabled comprehensive testing of consistency of the relationship between dental caries disease and treatment experience and dental fear in this sample of 16-year-olds residing in Malaysia.

Previous work in this area of dental caries disease and treatment experience and dental fear has been hampered by methodological weaknesses in the treatment of measurement error in the assessment of disease and psychological constructs. The use of maximum likelihood estimation within a structural equation framework has allowed a test of the simple disattentuated correlational model between the two latent constructs to our raw data. The generalizability of the strength of the relationship between DMFT and dental fear may be reasonably robust, even though this study is restricted to a single country, as this effect is less influenced by the mean levels of the various indicators that comprise these latent variables. Furthermore, in support of this opinion is that the level of fit was excellent as demonstrated by the non-significant chi square value for the overall test, and in addition the fit indices were well within recommended limits.

## Conclusions

A structural equation model was designed to inspect in detail the relationship between dental fear and dental caries disease and treatment experience with geography acting as a moderator. The finding has presented further evidence to support the relationship between dental caries disease and treatment experience with geography (locality) acting as a moderator. Future work requires development of more complex models to further understand the dynamics of dental fear and its possible influence on dental caries.

## Competing interests

The authors declare that there are no competing interests.

## Authors’ contributions

RE and OAL were the principal investigators of the study. RE, GH and RF have conceptualized and written the manuscript. GH has assisted with the statistical analyses and RF and GH have revised the manuscript. All authors read and approved the final manuscript.

## Pre-publication history

The pre-publication history for this paper can be accessed here:

http://www.biomedcentral.com/1472-6831/14/19/prepub
